# Longitudinal Associations Between Loneliness and Cognitive Ability in the Lothian Birth Cohort 1936

**DOI:** 10.1093/geronb/gby086

**Published:** 2018-07-21

**Authors:** Judith A Okely, Ian J Deary

**Affiliations:** Centre for Cognitive Ageing and Cognitive Epidemiology, Department of Psychology, University of Edinburgh, UK

**Keywords:** Cognition, Social interaction, Successful ageing

## Abstract

**Objectives:**

Loneliness is associated with poorer cognitive function in old age; however, the direction of this association is unknown. We tested for reciprocal associations between loneliness and the cognitive ability domains of processing speed, visuospatial ability, verbal memory, and crystallized ability.

**Method:**

We used three triennial waves of longitudinal data from the Lothian Birth Cohort Study 1936, and tested for cross-lagged associations between loneliness and cognitive abilities using cross-lagged panel models.

**Results:**

Better processing speed, visuospatial ability, or crystallized ability at age 73, was associated with less positive changes in loneliness between ages 73 and 76; however, these associations were not replicated between ages 76 and 79. Loneliness at ages 73 and 76 did not predict subsequent changes in cognitive abilities.

**Discussion:**

Our findings indicate an association between cognitive ability and loneliness, such that individuals with lower cognitive abilities at age 73 may be at a slightly higher risk of becoming lonely. However, we did not find support for the hypothesis that loneliness causes a decline in cognitive health.

Cognitive health—the ability to reason, think quickly, learn, and access stored knowledge—is a key determinant of older people’s quality of life and independence (Abrahamson, Clark, Perkins, & Arling, 2012; Tucker-Drob, 2011). Identifying the causes of cognitive decline, which is prevalent among older adults even without dementia (Boyle et al., 2013), is therefore a research priority. The experience of loneliness is recognized as a potential risk factor for cognitive decline, and has attracted particular attention, because loneliness and other social resource factors are potentially modifiable, and may therefore present targets for interventions to reduce cognitive ageing (Gow, Corley, Starr, & Deary, 2013).

Cognitive functioning can be divided into subdomains; these include: crystallized ability, which relates to learned knowledge and experience; processing speed, the speed with which mental processes are performed; visuospatial abilities, which include spatial visualization and the mental manipulation of objects; and memory. Although these abilities are conceptually distinct, there is a strong correlation in performance across domains of cognitive abilities: if an individual achieves a high score on tests of one cognitive ability domain, they are likely to perform well in other domains too (Deary, 2012).

Loneliness can be defined as the subjective experience of social isolation. It is distinct from objective social resource measures, such as marital status, social network size, or frequency of social contact, which have also been linked to cognitive ability and age-related cognitive decline (Kuiper et al., 2016; Thomas, 2011). Cross-sectional associations between greater loneliness and lower cognitive ability have been documented in several studies (Gilmour, 2011; Gow et al., 2013; Gow, Pattie, Whiteman, Whalley, & Deary, 2007), and longitudinal studies have reported an association between greater loneliness and steeper cognitive decline, which is partially independent of sociodemographic factors and some health conditions (Donovan et al., 2017; Gow & Mortensen, 2016; Shankar, Hamer, McMunn, & Steptoe, 2013; Wilson et al., 2007).

The direction of association between loneliness and cognitive health is not clear. On the one hand, it is possible that the experience of loneliness negatively influences cognitive ability; researchers have identified several possible mechanisms that could account for this direction of effect. For instance, the association between loneliness and cognitive ability could be mediated by subjective stress, which is a risk factor for dementia and cognitive decline (Aggarwal et al., 2014; Johansson et al., 2010). Loneliness is positively correlated with subjective stress (Cacioppo et al., 2006), and a heightened immune response following stressful experiences (Steptoe, Owen, Kunz-Ebrecht, & Brydon, 2004). Additionally, it is possible that loneliness or social isolation results in a lack of sensory and cognitive stimulation which, in turn, may increase the risk of cognitive decline (Wilson et al., 2007).

Although loneliness is generally considered to be a potential risk factor for cognitive decline, it is also possible that loneliness is caused by declining cognitive health (Donovan et al., 2017). Age-related cognitive decline is paralleled by a loss of independence and reduced social-cognitive functioning (Brown, Devanand, Liu, & Caccappolo, 2011; Washburn & Sands, 2006). These deficits may reduce an individual’s capacity to maintain friendships or participate in social activities, and increase the risk of social isolation and loneliness as a result.

Whereas previous studies have examined loneliness as a potential predictor of cognitive decline (Gow & Mortensen, 2016; Shankar et al., 2013; Wilson et al., 2007), few have considered the potentially reciprocal associations between cognitive ability and loneliness over time. One longitudinal study tested for this effect in a sample of 2,255 participants aged 55–85 years (Ellwardt, Aartsen, Deeg, & Steverink, 2013). The authors found evidence of a bidirectional effect whereby higher baseline levels of loneliness predicted steeper cognitive decline (*β* = −0.17), and lower baseline levels of cognitive ability predicted a greater increase in loneliness (*β* = −0.17). A second study, of 14,199 Chinese older adults, tested for reciprocal associations between loneliness and cognitive ability over a 9-year period (Zhong, Chen, Tu, & Conwell, 2017). Again, the authors found evidence of a bidirectional effect: higher loneliness was associated with a decrease in subsequent cognitive ability (*β* = −0.15), and higher cognitive ability was associated with a decrease in subsequent loneliness (*β* = −0.05).

The aim of the current study was to investigate further the interplay between loneliness and cognitive ability over most of the eighth decade of life. Using three triennial waves of longitudinal data from the Lothian Birth Cohort Study 1936 (LBC1936), we were able to build on previous work in several ways. First, our study provides a test of whether the previously documented bidirectional association between loneliness and cognitive ability can be replicated in a narrow-age cohort of Scottish older adults. Second, the studies described in the previous paragraph (Ellwardt et al., 2013; Zhong et al., 2017) used small cognitive test batteries. A limitation of such test batteries is that they may not be sensitive to subclinical changes in cognitive function (Ritchie et al., 2016). The extensive battery of cognitive tests in the LBC1936 allowed us to address this limitation and, additionally, test whether any longitudinal association between loneliness and cognitive ability is consistent across different domains of cognitive function. Finally, we were able to control for potentially mediating or confounding factors that were not included in previous studies (Ellwardt et al., 2013; Zhong et al., 2017), viz. childhood cognitive ability, depressive symptoms, and history of chronic disease.

## Methods

### Participants

The LBC1936 is a follow-up study of the Scottish Mental Survey of 1947, in which 70,805 children born in 1936 and attending Scottish schools completed a test of mental ability at a mean age of 11 years. (Scottish Council for Research in Education: The Trend of Scottish Intelligence, 1949). Surviving community-dwelling individuals born in 1936, most of whom took part in the Scottish Mental Survey of 1947, were recruited from the Edinburgh and Lothians areas of Scotland into the LBC1936 study; 1,091 people thus took part in the first wave of testing at a mean age of 70 years, between 2004 and 2007 (Deary et al., 2007; Taylor, Pattie, & Deary, 2018). Subsequent waves of testing were conducted from 2007–2010 (Wave 2), 2011–2013 (Wave 3), and 2014–2017 (Wave 4). Participants completed the same loneliness question at Waves 2, 3, and 4; therefore, we used data from these three waves in the current study. Eight hundred and sixty-six participants (mean age = 73, *SD* = 0.71) attended Wave 2, 697 (mean age = 76, *SD* = 0.68) attended Wave 3, and 550 participants (mean age = 79, *SD* = 0.62) attended Wave 4.

### Measures

#### Cognitive ability

Participants completed the same battery of 13 individually-administered cognitive tests at each wave of the study. As has been done previously with this data set (Ritchie et al., 2016), we grouped test scores according to 4 domains of cognitive ability: visuospatial ability, processing speed, verbal memory, and crystallized ability. Visuospatial ability was assessed with the Spatial Span (Forward and Backward) subtest from the Wechsler Memory Scale, 3rd UK Edition (Wechsler, 1998b), and the Matrix Reasoning and Block Design subtests from the Wechsler Adult Intelligence Scale, 3rd UK Edition (Wechsler, 1998a). Processing speed was assessed with a computer-based inspection time test, where participants must discriminate between two shapes which are backward masked following display times ranging from 200 ms to 6 ms (Deary et al., 2004); a four choice reaction time test, where participants press one of four buttons corresponding to the number displayed on a portable devise (Deary, Der, & Ford, 2001); and the Symbol Search and Digit-Symbol Substitution tests from the Wechsler Adult Intelligence Scale, 3rd UK Edition (Wechsler, 1998a). Verbal memory was measured with the Verbal Paired Associates and Logical Memory subtests from the Wechsler Memory Scale, 3rd UK Edition (Wechsler, 1998b), and the Digit Span Backward subtest from the Wechsler Adult Intelligence Scale, 3rd UK Edition (Wechsler, 1998a). Finally, crystallized ability was assessed with the National Adult Reading Test (NART; Nelson & Willison, 1991), the Wechsler Test of Adult Reading (Wechsler, 2001), and a test of phonemic verbal fluency (Lezak, 2004).

#### Loneliness

Loneliness was measured with a single item: “How much of the time during the past week have you felt lonely?” Response options were “none or almost none of the time”, “some of the time”, “most of the time” and “all or almost all of the time”. Because very few participants reported feeling lonely all or almost all the time (3, 4, and 2 participants at Waves 2, 3, and 4, respectively), we grouped these participants with those who reported feeling lonely most of the time. Loneliness was treated as an ordinal variable in the analysis.

#### Covariates

We adjusted for covariates previously associated with loneliness or cognitive ability that could confound or mediate this association. These were childhood cognitive ability, social class, symptoms of anxiety and depression, and history of chronic disease.

At age 11, LBC1936 participants completed the Moray House Test No. 12, a test of mainly verbal reasoning, as part of the Scottish Mental Survey of 1947 (Scottish Council for Research in Education, 1949). Participants’ scores were corrected for age in days at time of testing. Social class was indexed according to the Classification of Occupations (General, 1991) system, which consists of six classes ranging from professional to unskilled. Married women also reported their spouse’s social class and, if it was higher than their own, were assigned to that social class. Symptoms of depression and anxiety were assessed with the Hospital Anxiety and Depression scale (HADS; Zigmond & Snaith, 1983). Higher scores on this scale indicate higher symptoms of anxiety and depression. Participants were categorized as having a chronic disease if they reported a diagnosis of diabetes (type 1 or 2), cancer, stroke, or cardiovascular disease at age 73.

To test whether loneliness was associated with cognitive ability, independently of other social resource measures, we additionally included measures of marital status and social support at age 73. Participants reported their marital status as either: single, married, cohabiting, divorced, separated, or widowed. Social support was assessed with a 7-item scale previously used in the Health Survey for England (Grundy & Murphy, 2007). Possible scores range from 0 to 12 with higher scores indicating greater social support. Because this variable was highly skewed towards the maximum possible social support, it was recoded into a dichotomous variable (participants who reported maximum social support and participants who did not).

#### Analysis

The analysis was conducted in Mplus version 8 (Muthén, & Muthén, 2015) using the weighted least squares mean and variance adjusted estimator (WLSMV; (Muthén, & Muthén, 2015). The analytical sample included participants if they contributed cognitive or loneliness data at any of the three waves. With WLSMV estimation, the model is conditioned on the observed exogenous (i.e., independent) variables; cases with missing data on these variables are dropped from the analysis. Therefore, we excluded participants with missing data on covariate variables from our analytical sample. Seventy-six participants with missing covariate data were excluded. We also excluded participants with a Mini-Mental State Examination (MMSE; (Folstein, Folstein, & McHugh, 1975) score of less than 24 at any of the three waves. We excluded an additional 23 participants on this basis. The resulting analytical sample consisted of 767 participants. Of these participants, 620 contributed data at age 76 and 490 contributed data at age 79.

We used a cross-lagged panel modeling approach to test for reciprocal associations between loneliness and cognitive ability over time. Using this model, which measures interindividual change (Selig & Little, 2012), we were able to test whether an individual’s average relative standing on loneliness was related to subsequent changes in their average relative standing on cognitive ability, and conversely, whether prior average relative standing on cognitive ability predicted subsequent changes in average relative standing on loneliness.

We estimated separate models for each of the four cognitive domains (visuospatial ability, processing speed, verbal memory, and crystallized ability). Domains of cognitive ability were modeled as latent factors using the marker variable method of identification, whereby the first factor loading is fixed to 1. We specified equal factor loadings over time and autocorrelated measurement residuals. We also modeled residual correlations between concurrently-measured loneliness and cognitive ability factors (these residual correlations represent the shared effects of unobserved variables).

In Mplus, under WLSMV estimation with ordinal data, endogenous ordinal variables are transformed into continuous latent response variables using a probit transformation (Muthén, & Muthén, 2015). Path estimates from cognitive ability to subsequent levels of loneliness, represent the association between a SD increase in cognitive ability and the predicted probability of being in a higher loneliness category.

We ran two variants of these models: first, we adjusted loneliness and cognitive ability at age 73 for age and sex; and, second, we additionally adjusted loneliness and cognitive ability at age 73 for age 11 IQ, occupational social class, HADS score, history of chronic disease, marital status, and social support. Model fit was assessed with root mean square error of approximation (RMSEA) and weighted root mean square residual (WRMR). These fit indices are recommended for models with observed categorical variables (Newsom, 2018). RMSEA values <0.05 and WRMR below 1.0 indicate adequate model fit (Newsom, 2018).

Finally, we corrected *p*-values for multiple comparisons using Hochberg’s False Discovery Rate (FDR) correction (Benjamini & Hochberg, 1995). This correction was carried out separately for analysis with each of the cognitive ability subdomains. Results are reported with and without this correction.

## Results

Table 1 provides a summary of participant characteristics at each of the three waves. Mean scores on most of the cognitive tests decreased between ages 73 and 76, and between ages 76 and 79. The proportion of participants in each of the loneliness categories (never, some of the time, and most or all of the time) was similar at each wave, with most participants (between 82.2% and 82.9%) reporting never feeling lonely. The domains of cognitive ability were highly correlated. At age 73, these correlations ranged between *r* = .492, *p* < .001 (for processing speed and crystallized ability) and *r* = .693, *p* < .001 (for processing speed and visuospatial ability).

**Table 1.** Sample Characteristics at Ages 73, 76, and 79

	Age 73	Age 76	*p* ^a^	Age 79	*p* ^b^
*N*	767	620		490	
Matrix reasoning, *M* (*SD*)	13.36 (4.94)	13.25 (4.83)	.002	13.04 (4.94)	.052
Block design, *M* (*SD*)	34.01 (10.10)	32.70 (9.67)	<.001	31.54 (9.28)	<.001
Spatial span, *M* (*SD*)	7.41 (1.35)	7.35 (1.36)	.020	7.09 (1.33)	<.001
Paired associates, *M* (*SD*)	27.58 (9.30)	26.90 (9.30)	<.001	27.54 (9.31)	.218
Logical memory, *M* (*SD*)	75.32 (17.02)	75.98 (18.09)	.095	73.79 (19.34)	<.001
Digit span, *M* (*SD*)	7.89 (2.27)	7.87 (2.35)	.206	7.66 (2.14)	<.001
NART, *M* (*SD*)	34.83 (7.75)	35.52 (7.61)	.001	36.01 (7.75)	.486
WTAR, *M* (*SD*)	41.43 (6.49)	41.54 (6.59)	.120	42.02 (6.61)	.107
Verbal fluency, *M* (*SD*)	43.64 (12.84)	43.39 (12.80)	.016	44.21 (13.18)	.960
Digit Symbol, *M* (*SD*)	57.11 (12.07)	54.62 (12.57)	<.001	52.05 (12.44)	<.001
Symbol search, *M* (*SD*)	24.92 (6.04)	24.99 (6.18)	.026	22.96 (6.32)	<.001
Reaction time, *M* (*SD*)	0.64 (0.09)	0.67 (0.10)	<.001	0.70 (0.11)	<.001
Inspection time, *M* (*SD*)	111.49 (11.40)	110.55 (12.27)	<.001	107.27 (12.77)	<.001
Loneliness, *N* (%)			.789		.754
Never	635 (82.8)	503 (82.9)		396 (82.2)	
Some of the time	115 (15.0)	89 (14.7)		77 (16.0)	
Most or all the time	17 (2.2)	15 (2.5)		9 (1.9)	
Loneliness change, *N* (%)					
No change		513 (84.5)		385 (83.2)	
Lonelier		50 (8.2)		40 (8.6)	
Less lonely		44 (7.2)		38 (8.2)	
Married/cohabiting	545 (71.1)				
Maximum social support	470 (61.3)				
Female, *N* (%)	370 (48.2)				
HMSO social class, *N* (%)					
Professional	154 (20.1)				
Managerial/Technical	293 (38.2)				
Skilled nonmanual	171 (22.3)				
Skilled manual	124 (16.2)				
Partly skilled	21 (2.7)				
Unskilled	4 (0.5)				
Chronic disease, *N* (%)	359 (46.8)				
Age 11 IQ, *M* (*SD*)	101.32 (14.83)				
HADS, *M* (*SD*)	7.06 (4.43)				

*Note*: HADS = Hospital Anxiety and Depression scale; HMSO = Her Majesty’s Stationery Office; NART = National Adult Reading Test; WTAR = Wechsler Test of Adult Reading.

^a^ *p*-value for the significance of the difference between age 73 and 76. ^b^*p*-value for the significance of the difference between age 76 and 79.

Table 2 shows differences between participants who provided cognitive ability or loneliness data at all three waves (“completers”), *n* = 481, and those who provided data at only one or two waves (“noncompleters”), *n* = 286. Participants who completed all three waves of the study, had a higher occupational social class, a higher IQ at age 11, scored higher on all of the cognitive tests and reported fewer symptoms of anxiety and depression at age 73. Completers and noncompleters did not differ in terms of marital status, social support, loneliness, sex, or history of chronic disease at age 73. Supplementary Table 1 shows characteristics of participants, at ages 73, 76, and 79, who provided complete loneliness and cognitive ability data at all three waves, *n* = 364.

**Table 2.** Differences at Age 73 Between Participants Who Provided Data at All Three Waves (“completers”) and Participants Who Provided Data at One or Two Waves (“noncompleters”)

Characteristics at wave 2	Completers total *n* = 481	*n* ^a^	Noncompleters total *n* = 286	*n* ^b^	*p*
Matrix reasoning, *M* (*SD*)	14.04 (10.12)	479	12.20 (9.90)	285	<.001
Block design, *M* (*SD*)	34.94 (1.34)	480	32.44 (1.38)	285	.001
Spatial span, *M* (*SD*)	7.49 (8.75)	477	7.26 (9.82)	285	.022
Paired associates, *M* (*SD*)	28.85 (16.36)	471	25.44 (17.69)	279	<.001
Logical memory, *M* (*SD*)	77.15 (2.32)	481	72.21 (2.16)	284	<.001
Digit span, *M* (*SD*)	8.07 (7.62)	481	7.58 (7.81)	286	.003
NART, *M* (*SD*)	35.61 (6.24)	479	33.53 (6.76)	286	<.001
WTAR, *M* (*SD*)	42.11 (12.97)	479	40.30 (12.40)	286	<.001
Verbal fluency, *M* (*SD*)	44.80 (11.67)	480	41.70 (12.06)	286	.001
Digit symbol, *M* (*SD*)	59.04 (5.87)	478	53.88 (6.08)	286	<.001
Symbol search, *M* (*SD*)	25.73 (0.81)	478	23.57 (0.90)	285	<.001
Reaction time, *M* (*SD*)	0.63 (0.08)	480	0.66 (0.09)	286	<.001
Inspection time, *M* (*SD*)	112.47 (11.08)	472	109.77 (11.75)	271	.002
Loneliness, *N* (%)		481		286	.158
Never	407 (84.6)		228 (79.7)		
Some of the time	66 (13.7)		49 (17.1)		
Most or all the time	8 (1.7)		9 (3.1)		
Married/cohabiting, *N* (%)	339 (70.5)		206 (72.0)		.647
Maximum social support, *N* (%)	292 (60.7)		178 (62.2)		.674
Female, *N* (%)	236 (49.1)	481	134 (46.9)	286	.351
HMSO social class, *N* (%)		481		286	.002
Professional	116 (24.1)		38 (13.3)		
Managerial/Technical	189 (39.3)		104 (36.4)		
Skilled nonmanual	96 (20.0)		75 (26.2)		
Skilled manual	67 (13.9)		57 (19.9)		
Partly skilled	11 (2.3)		10 (3.5)		
Unskilled	2 (0.4)		2 (0.7)		
Chronic disease, *N* (%)	217 (45.1)	481	142 (49.7)	286	.223
Age 11 IQ, *M* (*SD*)	102.77 (14.58)	481	98.89 (14.94)	286	<.001
HADS, *M* (*SD*)	6.77 (4.25)	481	7.56 (4.69)	286	.019

*Note*: HADS = Hospital Anxiety and Depression scale; HMSO = Her Majesty’s Stationery Office; NART = National Adult Reading Test; WTAR = Wechsler Test of Adult Reading.

^a^Number of completers with available data at age 73. ^b^Number of noncompleters with available data at age 73.

**Figure 1.**
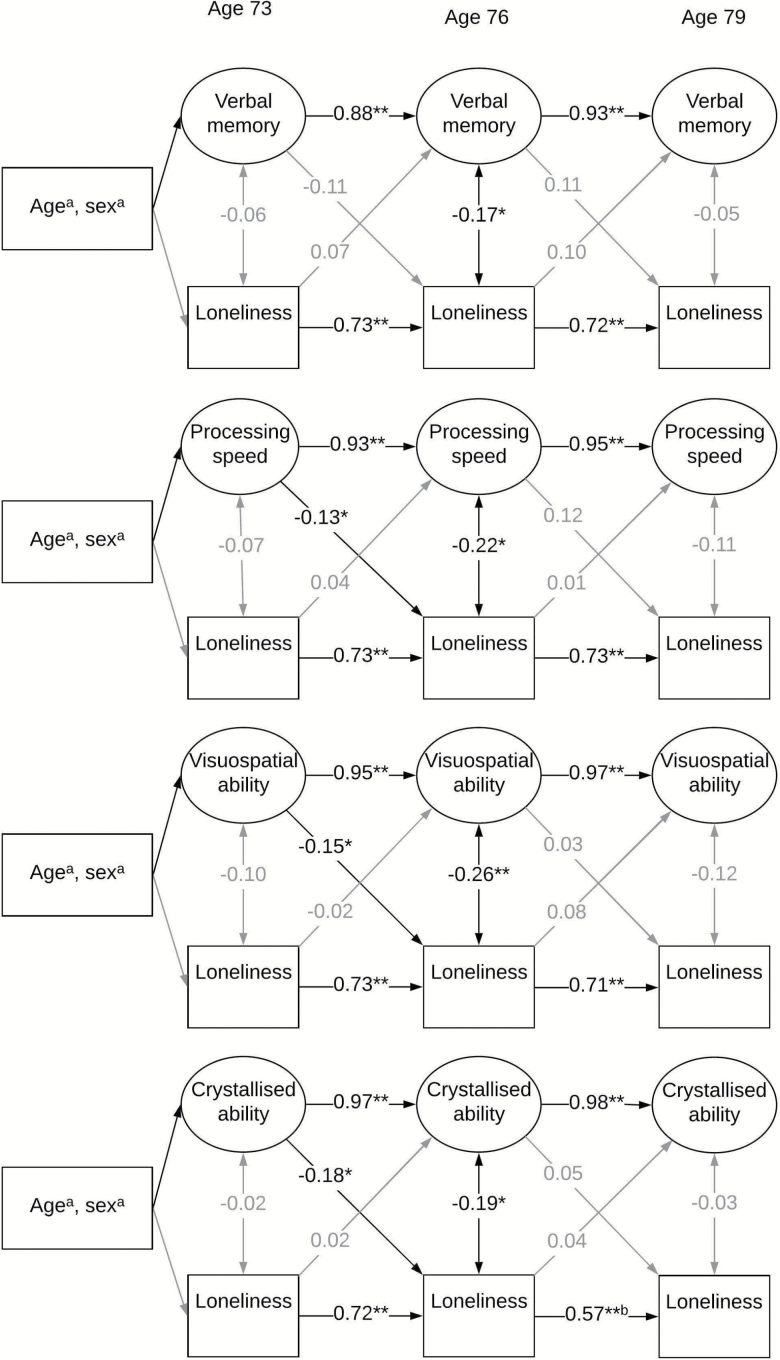
Age- and sex-adjusted standardized estimates from the cross-lagged panel models. Single headed arrows represent regression paths, double headed arrows represent correlations. Black arrows indicate paths significant at the *p* < .05 level. *p < .05, **p < 0.001. ^a^Covariates significantly associated with cognitive ability. ^b^Smaller coefficient estimate due to additional residual correlation between loneliness at age 76 and 79 in this model.

**Figure 2.**
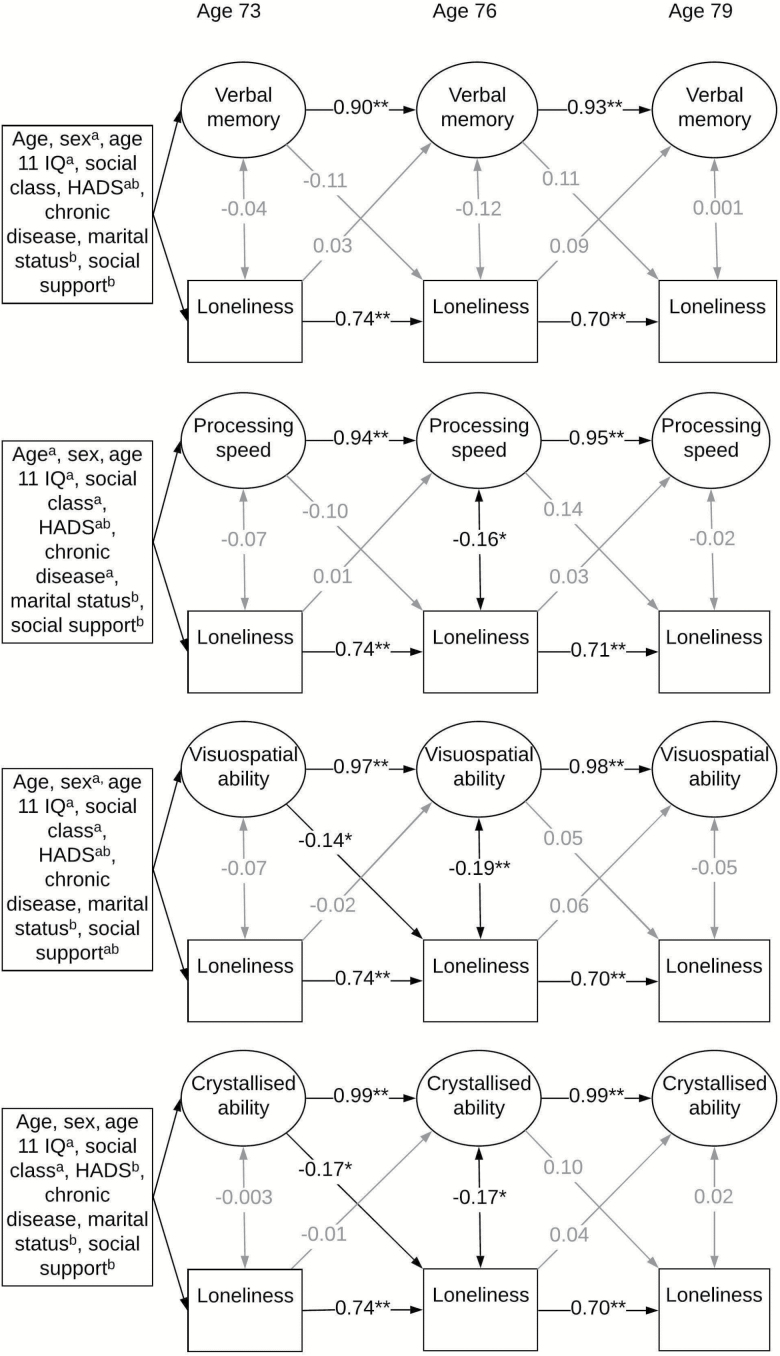
Standardized estimates from the cross-lagged panel models, additionally adjusted for age 11 IQ, occupational social class, Hospital Anxiety and Depression scale (HADS) score, history of chronic disease, marital status and social support. Single headed arrows represent regression paths, double headed arrows represent correlations. Black arrows indicate paths significant at the *p* < .05 level. *p < .05, **p < .001. ^a^Covariates significantly associated with cognitive ability. ^b^Covariates significantly associated with loneliness.

Figures 1 and 2 provide standardized estimates from the cross-lagged panel models. These estimates and their corresponding *p*-values are also displayed in Table 3. In the models, almost all wave-wave coefficients for cognitive domains were above 0.9. Most wave-wave coefficients for loneliness were around 0.7. These will not be described further. Fit indices for these models ranged within the acceptable levels: RMSEA = 0.013–0.038, WRMR = 0.493–0.894. Below we describe the contemporaneous and cross-lagged associations between loneliness and cognitive domains. Supplementary Table 2 shows the covariates which were significantly associated with cognitive ability domains or loneliness at age 73 in the fully-adjusted models.

**Table 3.** Standardized Estimates from the Cross-lagged Panel Models

Domain of cognitive ability	Model 1	*p*	Model 2	*p*
Verbal Memory				
Memory age 73 → memory age 76	**0.880**	<.001	**0.898**	<.001
Memory age 76 → memory age 79	**0.929**	<.001	**0.933**	<.001
Loneliness age 73 → loneliness age 76	**0.732**	<.001	**0.740**	<.001
Loneliness age 76 → loneliness age 79	**0.721**	<.001	**0.696**	<.001
Memory age 73 → loneliness age 76	-0.114	.104	-0.106	.156
Memory age 76 → loneliness age 79	0.110	.160	0.109	.195
Loneliness age 73 → memory age 76	0.066	.256	0.034	.517
Loneliness age 76 → memory age 79	0.095	.092	0.092	.083
Processing speed				
Speed age 73 → speed age 76	**0.932**	<.001	**0.940**	<.001
Speed age 76 → speed age 79	**0.950**	<.001	**0.945**	<.001
Loneliness age 73 → loneliness age 76	**0.730**	<.001	**0.741**	<.001
Loneliness age 76 → loneliness age 79	**0.727**	<.001	**0.705**	<.001
Speed age 73 → loneliness age 76	**-0.133**	.029	-0.099	.128
Speed age 76 → loneliness age 79	0.116	.138	0.144	.069
Loneliness age 73 → speed age 76	0.038	.432	0.001	.987
Loneliness age 76 → speed age 79	0.013	.801	0.025	.592
Visuospatial ability				
Visuospatial age 73 → visuospatial age 76	**0.947**	<.001	**0.971**	<.001
Visuospatial age 76 → visuospatial age 79	**0.972**	<.001	**0.978**	<.001
Loneliness age 73 → loneliness age 76	**0.728**	<.001	**0.736**	<.001
Loneliness age 76 → loneliness age 79	**0.713**	<.001	**0.699**	<.001
Visuospatial age 73 → loneliness age 76	**-0.146**	.024	**-0.144**	.048
Visuospatial age 76 → loneliness age 79	0.033	.662	0.046	.562
Loneliness age 73 → visuospatial age 76	0.022	.665	-0.002	.969
Loneliness age 76 → visuospatial age 79	0.084	.166	0.063	.249
Crystallized ability				
Crystallized age 73 → crystallized age 76	**0.973**	<.001	**0.990**	<.001
Crystallized age 76 → crystallized age 79	**0.980**	<.001	**0.992**	<.001
Loneliness age 73 → loneliness age 76	**0.721**	<.001	**0.740**	<.001
Loneliness age 76 → loneliness age 79	**0.567**	<.001	**0.700**	<.001
Crystallized age 73 → loneliness age 76	**-0.177**	.001	**-0.169**	.009
Crystallized age 76 → loneliness age 79	0.052	.448	0.101	.220
Loneliness age 73 → crystallized age 76	0.022	.543	-0.006	.815
Loneliness age 76 → crystallized age 79	0.043	.330	0.044	.121

*Note:* In model 1, loneliness and cognitive ability domain at age 73 are adjusted for age and sex. In model 2, loneliness and cognitive ability domain at age 73 are additionally adjusted for age 11 IQ, occupational social class, HADS score, history of chronic disease, marital status, and social support. Estimates in bold are significant at the 0.05 level.

### 

#### Verbal memory

In the age- and sex-adjusted model, none of the cross-lagged effects between loneliness and verbal memory were significant. The cross-sectional correlation between verbal memory and loneliness was nonsignificant at age 73 (*r* = −.063, *p* = .308) and age 79 (*r* = −.047, *p* = .536), but was significant at age 76 (*r =* −.170, *p* = .013). Estimates from the fully-adjusted model were largely similar to those from the age and sex adjusted model. However, the cross-sectional correlation between verbal memory and loneliness at age 76 was now nonsignificant.

#### Processing speed

Cross-lagged paths from loneliness to processing speed were non-significant in the age- and sex-adjusted model. We did observe a significant association between processing speed at age 73 and loneliness at age 76 (*β* = −0.133, *p* = .029, FDR corrected *p* = .046), such that participants with faster processing speed at age 73 were less likely to report higher levels of loneliness at age 76. Processing speed at age 76 was not associated with loneliness at age 79 (*β* = 0.116, *p* = .138). The cross-sectional correlation between processing speed and loneliness was nonsignificant at age 73 (*r* = −.071, *p* = .206) and age 79 (*r* = −.114, *p* = .096), but was significant at age 76 (*r =* −.219, *p* = .001); people with better processing speed scores reported being less lonely. The cross-lagged association between processing speed at age 73 and loneliness at age 76 was no longer significant in the fully adjusted model. The cross-sectional association between loneliness and processing speed at age 76 remained significant after adjustment (*r =* −.157, *p* = .015).

#### Visuospatial ability

All cross-lagged paths from loneliness to visuospatial ability were non-significant. Participants with higher visuospatial ability at age 73 were less likely to report higher levels of loneliness at age 76 (*β* = −0.146, *p* = .024, FDR corrected *p* = .038). Visuospatial ability at age 76 was not associated with loneliness at age 79 (*β* = 0.033, *p* = .662). The cross-sectional correlation between visuospatial ability and loneliness was nonsignificant at age 73 (*r* = −.097, *p* = .114) and age 79 (*r* = −.115, *p* = .117), but was significant at age 76 (*r =* −.256, *p* < .001); people with better visuospatial ability scores reported being less lonely. In the fully-adjusted model, the association between visuospatial ability at age 73 and loneliness at age 76 (*β* = −0.144, *p* = .048) did not survive FDR correction, with *p* = .077. In the fully-adjusted model, the cross-sectional association between visuospatial ability and loneliness at age 76 was *r* = −.191, *p* = .005.

#### Crystallized ability

For this model to converge, we also specified a residual correlation between loneliness at age 73 and age 79, and constrained the residual correlation between crystallized ability at age 73 and 76 to 1. None of the cross-lagged paths from loneliness to crystallized ability were significant. Participants with higher crystallized ability at age 73 were less likely to report higher levels of loneliness at age 76 (*β* = −0.177, *p* = .001, FDR corrected *p* = .002). The association between crystallized ability at age 76 and loneliness at age 79 was nonsignificant (*β* = 0.052, *p* = .448). The cross-sectional correlation between loneliness and crystallized ability was *r* = −.016 (*p =* .751) at age 73, *r* = −.187 (*p* = .001; people with better crystallized ability scores reported being less lonely) at age 76, and *r* = −.033 (*p* = .632) at age 79. In the fully-adjusted model, the association between crystallized ability at age 73 and loneliness at age 76 remained significant (*β* = −0.169, *p* = .009, FDR corrected *p* = .014), as did the cross-sectional correlation between crystallized ability and loneliness at age 76 (*r* = −.167, *p* = .010).

#### Probability values

Probit regression coefficients for ordinal data can be transformed into a probability value (Muthén & Muthén, 2015). In Table 4, we present the probability of being in the “sometimes lonely” category at age 76, for participants with a cognitive ability domain score 1 *SD* above or below the mean at age 73 (loneliness at age 73 was held constant at its mean for these calculations). Overall, the probability of being in the sometimes lonely category at age 76 was low. However, on average, the probability of being in this category was almost twice as high for participants with a cognitive ability domain score 1 *SD* below the mean compared to participants with a cognitive ability domain score 1 *SD* above the mean.

**Table 4.** The Predicted Probability of Being in the “Sometimes Lonely” Category at Age 76 Associated with a Cognitive Ability Domain Score 1 *SD* Above or Below the Mean at Age 73

Domain	Cognitive ability score at age 73	Probability of being in the “sometimes lonely” category at age 76
Processing speed	+1 *SD*	0.092 (95% CI: 0.060–0.135)
	−1 *SD*	0.174 (95% CI: 0.142–0.209)
Visuospatial ability	+1 *SD*	0.106 (95% CI: 0.091–0.122)
	−1 *SD*	0.205 (95% CI: 0.130–0.295)
Crystallized ability	+ 1 *SD*	0.077 (95% CI: 0.050–0.114)
	−1 *SD*	0.181 (95% CI: 0.153–0.211)

*Note:* Estimates from analysis adjusted for age and sex. CI = confidence interval.

#### Sensitivity analysis

To test whether excluding participants with an MMSE score of less than 24 influenced our results, we reran the age- and sex-adjusted models including the 23 participants who were excluded from the original sample on this basis. Results from the models with verbal memory, visuospatial ability, and crystallized ability were very similar to those from the original analysis. Contrary to our original findings, the cross-lagged association between processing speed at age 73 and loneliness at age 76 was nonsignificant (*β* = −0.114, *p* = .057). Our original estimate of this association was *β* = −0.133, *p* = .029. See Supplementary Table 3 for these results.

At each wave of the study, approximately 2% of the participants reported feeling lonely most or all of the time, whereas approximately 83% reported feeling lonely none or almost none of the time. To test whether the skewed distribution of the loneliness variable affected our results, we reran the age- and sex-adjusted models using a dichotomized version of the loneliness variable: lonely none or almost none of the time versus lonely some of the time, most of the time or all of the time. Estimates were similar to those from the original analysis, we observed a significant negative association between processing speed, visuospatial ability and crystallized ability at age 73 and levels of loneliness at age 76. The association between verbal memory and loneliness remained nonsignificant. See Supplementary Table 3 for these results.

## Discussion

The direction of association between loneliness and cognitive ability remains to be established. In this study, we used data from a narrow age cohort of community dwelling older adults to test for reciprocal associations between loneliness and cognitive abilities. We found that better processing speed, visuospatial ability, or crystallized ability at age 73, was associated with less positive changes in loneliness between ages 73 and 76; however, these associations were not replicated between ages 76 and 79. We did not find evidence of the potentially deleterious effect of loneliness on cognitive health: loneliness at ages 73 and 76 did not predict subsequent changes in cognitive ability.

Our finding of a one-directional path from cognitive ability at age 73 to changes in loneliness between ages 73 and 76, suggests that the association between cognitive ability and loneliness might be caused by health selection processes. Age-related cognitive decline can result in a loss of independence and a reduction in social-cognitive functioning (Brown et al., 2011; Washburn & Sands, 2006), which in turn, may increase the risk of social isolation and loneliness over time. A decline in social-cognitive abilities, which include detecting and responding to social cues and other people’s emotions (Henry, Von Hippel, Molenberghs, Lee, & Sachdev, 2016), could affect an individual’s evaluation of their relationships as well as the quality of their social interactions.

Our results are not consistent with those reported by previous studies into associations between cognitive ability and loneliness (Ellwardt et al., 2013; Zhong et al., 2017). These two studies report bidirectional effects between loneliness and cognitive ability. It is unclear why we failed to detect such an effect. However, the studies by Ellwardt et al. (2013) and Zhong et al. (2017) had larger samples sizes (*n* = 2,255 and *n* = 4,199, respectively) than our study (*n* = 767). It is possible that our analysis lacked power to detect the weak effect of loneliness on cognitive decline. Additionally, the loneliness measures used by Ellwardt et al. (2013) and Zhong et al. (2017), were different, and, in the case of the study by Ellwardt et al. (2013), more comprehensive than the loneliness measure used here. It is likely that studies with more comprehensive measures of loneliness (e.g., the De Jong Gierveld Loneliness Scale [de Jong-Gierveld & Kamphuls, 1985)] or the UCLA loneliness scale [Russell, 1996]) are better able to detect associations between loneliness and subsequent cognitive decline.

Although we found evidence that cognitive ability predicts changes in loneliness, this effect was not consistent across domains of cognitive ability or measurement occasions. Our results suggest that the domains of crystallized ability, visuospatial ability and processing speed are more closely related loneliness than is verbal memory. Crystallized abilities are typically acquired in social environments such as work or school (Cattell, 1987); thus, individuals with higher crystallized abilities may have a acquired more social resources and be less at risk of becoming lonely as a result. In addition, previous work has illustrated that fluid cognitive abilities (as indexed by visuospatial reasoning in our study) and processing speed are critical for sociocognitive processes including the processing of social cues and regulating emotional responses (Doerwald, Scheibe, Zacher, & Van Yperen, 2016). However, there is also evidence that memory contributes to these functions (Doerwald et al., 2016). Thus, it is unclear why we did not detect an association between verbal memory and loneliness in our study. One possibility, is that only memory deficits more severe than those present in our sample negatively impact sociocognitive processes related to loneliness.

Although processing speed, visuospatial ability, and crystallized ability at age 73 predicted change in loneliness between ages 73 and 76, these associations were not replicated between ages 76 and 79. This inconsistency could indicate that the relationship between these factors varies as a function of age, with a stronger relationship at younger ages; however, further work is needed to verify this. Alternatively, it is possible that our study was underpowered to detect associations between cognitive abilities and loneliness at later stages of the study, when the sample size—and the number of lonely participants—was reduced.

In addition to the cross-lagged associations between cognitive abilities and subsequent experiences of loneliness, we found evidence of a cross-sectional association between these variables at age 76. The temporal sequence of change in loneliness and cognitive abilities in later life is not yet clear. It is possible that these factors change concurrently (as suggested by the cross-sectional association observed here), or within closer temporal proximity than we could model in our study, in which loneliness and cognitive abilities were assessed on a triennial basis.

Strengths of our study included the extensive battery of cognitive tests, which allowed us to test for associations between loneliness and subdomains of cognitive ability; and, the fact that we could adjust for a range of potentially mediating or confounding variables rarely included in previous studies. One limitation of our study is that loneliness was measured with a single item, it is likely that this item was less sensitive than more comprehensive measures of loneliness. Furthermore, in our sample, participants’ relative standing on levels of cognitive ability and loneliness changed little over time (as indicated by the large autoregressive coefficients). The stability of loneliness and cognitive abilities—which are notable empirical findings in themselves—may have reduced our chance of detecting any longitudinal associations between these variables. Participant attrition, although not related to loneliness, was associated with lower cognitive ability, as well as lower age 11 IQ, occupational social class and higher symptoms of anxiety and depression. The reduced variance in cognitive ability resulting from this selective attrition may have biased our results toward the null. The association between attrition and symptoms of anxiety and depression may have introduced a further source of bias. Depressive symptoms are positively associated with loneliness (Erzen & Çikrikci, 2018); thus, it is possible that participants at a higher risk of becoming lonely were less likely to remain in the study. Finally, limitations relating to the cross-lagged panel model should be acknowledged. This model describes change in average relative standing on the measured variables, but is not sensitive to trajectories of within person change (Selig & Little, 2012). Hamaker, Kuiper, & Grasman (2015) have developed an alternative to the traditional cross-lagged panel model, for continuous data, which distinguishes between within and between person levels of change. However, an equivalent model for ordinal data is not yet available.

Policy makers and charities have increasingly recognized the importance of tackling the issue of loneliness among older adults (Newall & Menec, 2017). Our findings suggest that those who experience age-related cognitive decline may be at slightly increased risk of becoming lonely, and may therefore particularly benefit from interventions designed to reduce social isolation. However, our findings do not support the theory that loneliness has detrimental consequences for cognitive health. Further research exploring this latter association is needed. Particularly, researchers should seek to identify the life-stage(s) at which this association is most apparent, and crucially, whether interventions for loneliness contribute to a reduced risk of cognitive decline.

## Supplementary Material

Supplementary data is available at *The Journals of Gerontology, Series B: Psychological Sciences and Social Sciences* online.

gby086_suppl_Supplemetary_File

## Funding

LBC1936 data collection was supported by Age UK (Disconnected Mind project). The work was undertaken in The University of Edinburgh Centre for Cognitive Ageing and Cognitive Epidemiology, which is supported by funding from Biotechnology and Biological Sciences Research Council (BBSRC) and the Medical Research Council (MRC) as part of the cross council Lifelong Health and Wellbeing Initiative (grant number MR/K026992/1).

## Conflict of Interest

None reported.
