# Supplementary material for: Longitudinal Associations Between Loneliness and Cognitive Ability in the Lothian Birth Cohort 1936
Source: J Gerontol B Psychol Sci Soc Sci. 2018 Jul 21;74(8):1376–86. doi: 10.1093/geronb/gby086 (PMC6777773; doi:10.1093/geronb/gby086)
Supplement: gby086_suppl_Supplemetary_File [file gby086_suppl_supplemetary_file.docx]

| Supplementary table 1  *Characteristics of participants, at ages 73, 76 and 79, who provided complete loneliness and cognitive data at all three waves, n = 364* | | | | | |
| --- | --- | --- | --- | --- | --- |
|  | Age 73 | Age 76 | *P^a^* | Age 79 | *P^b^* |
| Matrix reasoning, *M* (SD) | 14.22 (4.75) | 13.78 (4.69) | .041 | 13.39 (4.83) | .069 |
| Block design, *M* (SD) | 35.37 (10.09) | 33.49 (9.56) | <.001 | 31.97 (9.20) | <.001 |
| Spatial span, *M* (SD) | 7.58 (1.35) | 7.49 (1.35) | .154 | 7.18 (1.34) | <.001 |
| Verbal associates, *M* (SD) | 29.43 (8.47) | 28.26 (8.63) | .001 | 27.98 (8.97) | .411 |
| Logical memory, *M* (SD) | 78.69 (15.01) | 78.78 (15.97) | .882 | 76.01 (17.15) | <.001 |
| Digit span, *M* (SD) | 8.24 (2.31) | 8.12 (2.44) | .221 | 7.83 (2.18) | .004 |
| NART, *M* (SD) | 35.74 (7.58) | 36.26 (7.39) | <.001 | 36.27 (7.55) | .906 |
| WTAR, *M* (SD) | 42.32 (6.30) | 42.28 (6.35) | .778 | 42.40 (6.50) | .361 |
| Verbal fluency, *M* (SD) | 45.75 (13.05) | 45.61 (12.65) | .730 | 45.54 (12.76) | .872 |
| Digit symbol, *M* (SD) | 60.02 (11.31) | 57.29 (11.45) | <.001 | 53.57 (11.70) | <.001 |
| Symbol search, *M* (SD) | 26.21 (5.57) | 26.36 (5.70) | .543 | 23.60 (6.03) | <.001 |
| Reaction time, *M* (SD) | 0.63 (0.08) | 0.66 (0.09) | <.001 | 0.69 (0.10) | <.001 |
| Inspection time, *M* (SD) | 112.60 (11.39) | 111.76 (11.36) | .125 | 107.89 (12.68) | <.001 |
| Loneliness, *N* (%) |  |  | .555 |  | .554 |
| Never | 310 (85.2) | 307 (84.3) |  | 301 (82.7) |  |
| Some of the time | 51 (14.0) | 50 (13.7) |  | 59 (16.2) |  |
| Most or all the time | 3 (0.8) | 7 (1.9) |  | 4 (1.1) |  |
| Loneliness change, *N* (%) |  |  |  |  |  |
| No change |  | 306 (84.1) |  | 300 (82.4) |  |
| Lonelier |  | 32 (8.8) |  | 34 (9.3) |  |
| Less lonely |  | 26 (7.1 |  | 30 (8.2) |  |
| ^a^ p-value for the significance of the difference between age 73 and 76.  ^b^ p-value for the significance of the difference between age 76 and 79. | | | | | |

| Supplementary table 2  *Standardised estimates for the covariates from fully adjusted models of loneliness and verbal memory, processing speed, visuospatial reasoning or crystallised ability* | | | |
| --- | --- | --- | --- |
| Domain of cognitive ability | *β* | S.E. | *p* |
| **Verbal memory** |  |  |  |
| Age | -0.060 | 0.039 | .130 |
| Sex: female vs. male | 0.309 | 0.093 | .001 |
| Age 11 IQ | 0.558 | 0.037 | <.001 |
| History of chronic disease: reported vs. not | 0.114 | 0.081 | .160 |
| HADS score | -0.089 | 0.040 | .026 |
| Occupational social class: |  |  |  |
| Managerial/Technical vs. professional | -0.151 | 0.108 | .162 |
| Skilled non-manual vs. professional | -0.098 | 0.128 | .444 |
| Skilled manual vs. professional | -0.175 | 0.148 | .237 |
| Partly skilled/unskilled vs. professional | -0.150 | 0.255 | .557 |
| Marital status: married/cohabiting vs. single | 0.005 | 0.092 | .957 |
| Social support: maximum vs. less than maximum | -0.006 | 0.087 | .948 |
| **Processing speed** |  |  |  |
| Age | -0.134 | 0.036 | <.001 |
| Sex: female vs. male | -0.058 | 0.081 | .471 |
| Age 11 IQ | 0.378 | 0.037 | <.001 |
| History of chronic disease: reported vs. not | -0.178 | 0.072 | .014 |
| HADS score | -0.153 | 0.035 | <.001 |
| Occupational social class: |  |  |  |
| Managerial/Technical vs. professional | -0.207 | 0.097 | .033 |
| Skilled non-manual vs. professional | -0.099 | 0.113 | .382 |
| Skilled manual vs. professional | -0.673 | 0.128 | <.001 |
| Partly skilled/unskilled vs. professional | -0.671 | 0.241 | .005 |
| Marital status: married/cohabiting vs. single | -0.054 | 0.082 | .510 |
| Social support: maximum vs. less than maximum | 0.096 | 0.076 | .208 |
| **Visuospatial reasoning** |  |  |  |
| Age | -0.049 | 0.036 | .165 |
| Sex: female vs. male | -0.548 | 0.079 | <.001 |
| Age 11 IQ | 0.464 | 0.035 | <.001 |
| History of chronic disease: reported vs. not | -0.130 | 0.072 | .071 |
| HADS score | -0.107 | 0.037 | .004 |
| Occupational social class: |  |  |  |
| Managerial/Technical vs. professional | -0.310 | 0.097 | .001 |
| Skilled non-manual vs. professional | -0.362 | 0.114 | .001 |
| Skilled manual vs. professional | -0.592 | 0.124 | <.001 |
| Partly skilled/unskilled vs. professional | -0.741 | 0.200 | <.001 |
| Marital status: married/cohabiting vs. single | 0.049 | 0.082 | .552 |
| Social support: maximum vs. less than maximum | 0.229 | 0.074 | .002 |
| **Crystallised ability** |  |  |  |
| Age | -0.050 | 0.029 | .081 |
| Sex: female vs. male | -0.012 | 0.066 | .856 |
| Age 11 IQ | 0.636 | 0.021 | <.001 |
| History of chronic disease: reported vs. not | -0.039 | 0.055 | .487 |
| HADS score | 0.005 | 0.028 | .851 |
| Occupational social class: |  |  |  |
| Managerial/Technical vs. professional | -0.186 | 0.083 | .025 |
| Skilled non-manual vs. professional | -0.391 | 0.094 | <.001 |
| Skilled manual vs. professional | -0.645 | 0.092 | <.001 |
| Partly skilled/unskilled vs. professional | -0.561 | 0.186 | .003 |
| Marital status: married/cohabiting vs. single | -0.073 | 0.063 | .248 |
| Social support: maximum vs. less than maximum | 0.021 | 0.058 | .720 |
| **Lonelines**^a^ |  |  |  |
| Age | -0.003 | 0.048 | .954 |
| Sex: female vs. male | 0.033 | 0.104 | .753 |
| Age 11 IQ | 0.029 | 0.054 | .588 |
| History of chronic disease: reported vs. not | 0.112 | 0.092 | .224 |
| HADS score | 0.441 | 0.038 | <.001 |
| Occupational social class: |  |  |  |
| Managerial/Technical vs. professional | -0.138 | 0.126 | .272 |
| Skilled non-manual vs. professional | -0.200 | 0.145 | .166 |
| Skilled manual vs. professional | 0.169 | 0.159 | .287 |
| Partly skilled/unskilled vs. professional | 0.247 | 0.302 | .414 |
| Marital status: married/cohabiting vs. single | -0.687 | 0.088 | <.001 |
| Social support: maximum vs. less than maximum | -0.382 | 0.091 | <.001 |
| ^a^ Estimates for the association between covariate variables and loneliness are taken from the model of loneliness and processing speed. These estimates were very similar in the models with the other three cognitive ability domains. | | | |

| Supplementary table 3  *Standardised estimates from the original cross-lagged panel models (model 1) and from the two sensitivity analyses: including participants with an MMSE score of less than 24 (model 2), and using a dichotomised version of the loneliness variable (model 3).* | | | | | | |
| --- | --- | --- | --- | --- | --- | --- |
| Domain of cognitive ability | Model 1  N = 767 | *p* | Model 2  N = 790 | *p* | Model 3  N = 767 | *p* |
| Verbal Memory |  |  |  |  |  |  |
| memory age 73 →  loneliness age 76 | -0.114 | .104 | -0.118 | .074 | -0.107 | .147 |
| memory age 76 →  loneliness age 79 | 0.110 | .160 | 0.083 | .268 | 0.101 | .215 |
| loneliness age 73 →  memory age 76 | 0.066 | .256 | 0.070 | .148 | 0.084 | .151 |
| loneliness age 76 →  memory age 79 | 0.095 | .092 | 0.088 | .100 | 0.105 | .067 |
| Processing speed |  |  |  |  |  |  |
| speed age 73 →  loneliness age 76 | **-0.133** | .029 | -0.114 | .057 | **-0.132** | .036 |
| speed age 76 →  loneliness age 79 | 0.116 | .138 | 0.096 | .207 | 0.104 | .201 |
| loneliness age 73 →  speed age 76 | 0.038 | .432 | 0.054 | .258 | 0.046 | .330 |
| loneliness age 76 →  speed age 79 | 0.013 | .801 | 0.010 | .838 | 0.026 | .623 |
| Visuospatial ability |  |  |  |  |  |  |
| visuospatial age 73 →  loneliness age 76 | **-0.146** | .024 | **-0.145** | .021 | **-0.129** | .049 |
| visuospatial age 76 →  loneliness age 79 | 0.033 | .662 | 0.019 | .795 | 0.038 | .626 |
| loneliness age 73 →  visuospatial age 76 | 0.022 | .665 | 0.028 | .571 | 0.033 | .514 |
| loneliness age 76 →  visuospatial age 79 | 0.084 | .166 | 0.068 | .242 | 0.094 | .118 |
| Crystallised ability |  |  |  |  |  |  |
| crystallised age 73 →  loneliness age 76 | **-0.177** | .001 | **-0.168** | .001 | **-0.165** | .002 |
| crystallised age 76 →  loneliness age 79 | 0.052 | .448 | 0.017 | .790 | 0.052 | .491 |
| loneliness age 73 →  crystallised age 76 | 0.022 | .543 | 0.027 | .435 | 0.020 | .583 |
| loneliness age 76 →  crystallised age 79 | 0.043 | .330 | 0.031 | .439 | 0.053 | .223 |
| *Note.* All estimates are adjusted for age and sex only. | | | | | | |
